# The Transactions of the American Medical Association, Vol. IV

**Published:** 1852-02

**Authors:** 


*The Transactions of the American Medical Association, Vol. IV.*— The Transactions for the present year, make a volume of a little less than seven hundred pages. The Report on Medical Sciences extends over forty-four pages. In this Report Dr. Dowler gives a succinct account of American contributions during the year relating to Anatomy and Physiology, Medical Chemistry, Pharmacy, Pathology, etc. Appended to the Report are three papers, one containing an account of a remarkable case of Amaurosis, illustrating the anatomy of the optic nerves, by Henry F. Campbell, M. D., Demonstrator of Anatomy in the Medical College of Georgia; a report of a committee of the Physico-Medical Society of New Orleans on lead poisoning, and Miscellaneous Memoranda, by the chairman of the committee.

The Report on Practical Medicine occupies a little less than one hundred pages. Appended to this Report are, *first,* a valuable paper, giving an account of Cholera in Cincinnati during the year 1850, by George Mendenhall, M. D.; *second,* an able report on the Fevers, etc. of New Orleans, by E. D. Fenner, M. D.; *third,* a descriptive account of the epidemic fever called Dergue, as it prevailed at Charleston, S. C., and Augusta, Ga., compiled by the chairman of the committee, from papers contributed by Prof. Dickson and Dr. Wragg, of Charleston, and Dr. Campbell, of Augusta; *fourth,* an account of an epidemic which prevailed in Allegany Co., N. Y., during the autumn and winter of 1850 – 51, by Dr. R. P. Stevens.

The Report on Surgery, by Prof. Paul F. Eve, chairman, covers thirty-five pages. This, like the other reports, is followed by an appendix containing valuable contributions. Dr. Buck furnishes an account of six additional cases of Œdematous Laryngitis, treated successfully by scarifications of the epiglottis and anyteno-epiglottic folds. Dr. Atlee contributes a table of all the known operations of Ovariotamy from 1701 to 1851, comprising 222 cases, and giving a synoptical history of each case. Dr. Lente gives the surgical statistics of the New York Hospital, compiled from the hospital records, at the request of Dr. John Watson, attending surgeon.

The Report on Obstetrics, D. Humphreys Storer, M. D., chairman, extends over fifty pages.

The Report on Medical Education, Worthington Hooker, M. D., chairman, occupies about thirty pages. To this report are appended a distinct report on Demonstrative Midwifery, and a communication from Prof. Horsford, of Harvard University, on the subject of instruction in Practical Chemistry.

The Report on Medical Literature, Thos. Reyburn, M. D., chairman, about sixty pages. This is followed by the Report of the committee on publication, Isaac Hays, M. D., chairman.

The last of the Reports is on Hygiene, P. C. Gaillard, M. D., chairman, extending over twenty-eight pages.

The remainder of the volume is devoted to the prize essay on the Corpus Luteum of menstruation and pregnancy, by Prof. John C. Dalton. This essay covers about one hundred pages.

We must content ourselves, for the present, with the foregoing catalogue of contents of this volume of the Transactions. We shall notice some of the Reports at more or less length hereafter.

We learn with regret that at the large and disastrous fire which recently occurred at Philadelphia, two-thirds of the edition of the fourth volume of the Transactions was consumed, and nearly all the previous volumes remaining on hand. This is a great loss to the Association and to the medical profession of this country. Members who have paid their dues will be furnished with copies, if not already supplied, and the remainder will be sold at an advanced price.

A few copies of the prize essay, by Prof. Dalton, were preserved, and will be for sale. This is an able production, based on original observations tending to establish points of distinction between the corpus luteum of pregnancy, and that of menstruation. This distinction, which is one not only of scientific interest, but possessing important medico-legal relations, may be considered to be settled by the observations of Prof. D. The essay embraces beautifully colored lithographic plates from drawings by the author of the essay. We advise our readers not to delay in securing copies of this work.

---

*A good opening for a Physician!* —A correspondent of the Philadelphia Medical Examiner, writing from Selma, Alabama, says, "we have in this pleasant little town, situated on the Alabama river 300 miles above Mobile, a population of about fifteen hundred inhabitants, white and black, young and old, and *fifteen regular practitioners of medicine.*"

